# The Wuerzburg procedure: the tensor fasciae latae perforator is a reliable anatomical landmark to clearly identify the Hueter interval when using the minimally-invasive direct anterior approach to the hip joint

**DOI:** 10.1186/s12891-016-0908-z

**Published:** 2016-02-03

**Authors:** Maximilian Rudert, Konstantin Horas, Maik Hoberg, Andre Steinert, Dominik Emanuel Holzapfel, Stefan Hübner, Boris Michael Holzapfel

**Affiliations:** Department of Orthopaedic Surgery, University of Wuerzburg Koenig-Ludwig Haus, Brettreichstr. 11, 97074 Wuerzburg, Germany; Dapartment of Trauma, Orthopaedics, Hand and Reconstructive Surgery, Clinic Harlaching, Sanatoriumsplatz 2, 81545 Munich, Germany; Institute of Anatomy and Cell Biology, University of Wuerzburg, Koellikerstr. 6, 97070 Wuerzburg, Germany; Regenerative Medicine, Institute of Health and Biomedical Innovation, Queensland University of Technology, 60 Musk Avenue, Kelvin Grove, QLD 4049 Brisbane, Australia

**Keywords:** Direct anterior approach, Hueter interval, Minimally-invasive, Hip replacement, Perforator, Anatomical landmark

## Abstract

**Background:**

The key for successful delivery in minimally-invasive hip replacement lies in the exact knowledge about the surgical anatomy. The minimally-invasive direct anterior approach to the hip joint makes it necessary to clearly identify the tensor fasciae latae muscle in order to enter the Hueter interval without damaging the lateral femoral cutaneous nerve. However, due to the inherently restricted overview in minimally-invasive surgery, this can be difficult even for experienced surgeons.

**Methods and Surgical Technique:**

In this technical note, we demonstrate for the first time how to use the tensor fasciae latae perforator as anatomical landmark to reliably identify the tensor fasciae latae muscle in orthopaedic surgery. Such perforators are used for flaps in plastic surgery as they are constant and can be found at the lateral third of the tensor fasciae latae muscle in a direct line from the anterior superior iliac spine.

**Conclusion:**

As demonstrated in this article, a simple knowledge transfer between surgical disciplines can minimize the complication rate associated with minimally-invasive hip replacement.

## Background

**Fig. 1:**
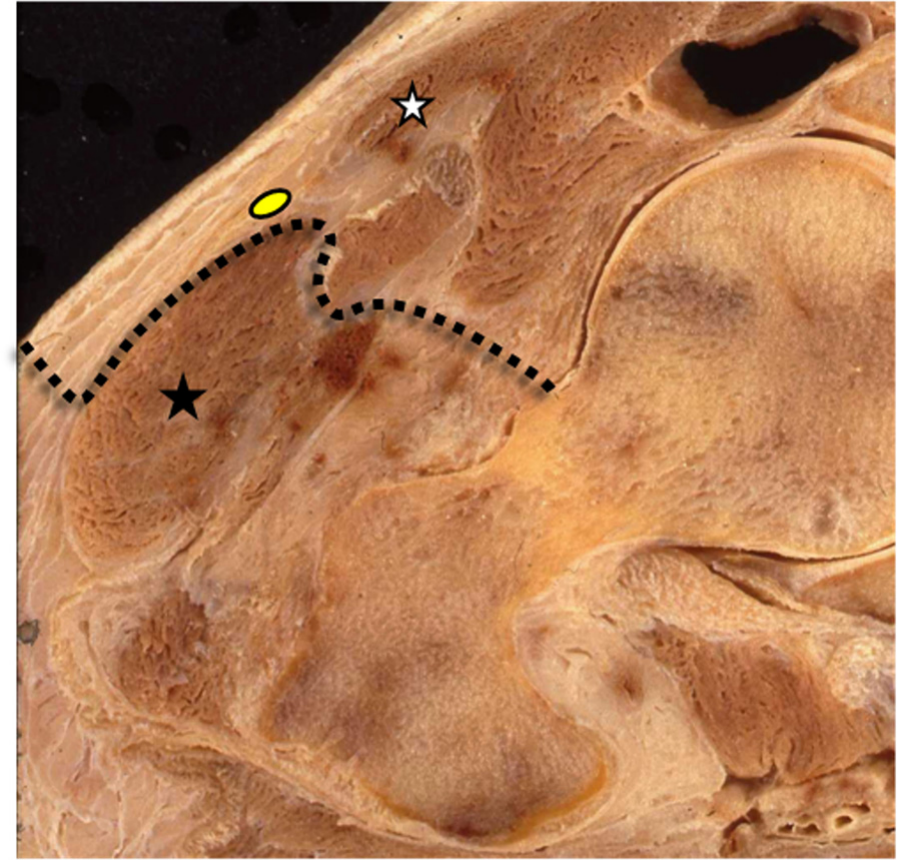
Axial cross-section through the hip joint and its surrounding soft tissues: The dotted line indicates the surgical approach with lateral incision of the superficial thigh fascia above the belly of the tensor and subsequent blunt dissection to enter the Hueter interval between the tensor fasciae latae muscle (*black asterisk*) and the sartorius muscle (*white asterisk*). This approach allows the protection of the main trunk of the lateral femoral cutaneous nerve (*yellow circle*) leaving it untouched within the fascial tunnel

Several anatomical landmarks have been proposed for the reliable identification of minimally-invasive surgical approaches in total hip arthroplasty [1–3]. The correct identification of such landmarks is a *conditio sine qua non* to avoid complications related to the restricted overview of the surgical situs [4]. An elaborate description of the surgical anatomy of the anterior approach to the hip joint was first published by Carl Hueter in 1881 [5]. In 1917 Marius Smith-Peterson was the first to propagate the use of this approach throughout the English-speaking surgical community [6]. Since then multiple other authors developed minimally-invasive techniques and continued to use those in various modifications to treat a *plethora* of different disorders around the hip joint [7–11]. All those methods have in common that they utilise the muscular interval between the sartorius and the tensor fasciae latae muscle, which is known as the Hueter interval. Using this interval poses the risk of damaging the lateral femoral cutaneous nerve as its main trunk usually runs along the medial border of the proximal tensor fasciae latae muscle. At this location, the nerve is covered by the superficial thigh fascia in approximately 90 % of the cases [12]. To minimise the risk for iatrogenic nerve injury, some authors advocated an elegant technique where they incise the superficial thigh fascia as laterally as possible over the belly of the tensor fasciae latae muscle followed by blunt dissection between the muscle and the superficial fascia thereby entering the Hueter interval [13–15] (Fig. 1). By doing so, the nerve and its branches can stay untouched, embedded in the superficial thigh fascia. However, this technique makes it necessary to clearly identify the tensor fasciae latae muscle and its overlaying fascia. Due to the restricted overview in minimally-invasive surgery, this can be challenging even for experienced surgeons [11]. The unambiguous identification of the muscle can furthermore be hindered by a thick subcutaneous fat layer or an inadequate positioning of the skin incision. We therefore propose the use of a constant anatomical landmark that makes it possible to clearly identify the superficial thigh fascia and the tensor muscle.

### Surgical technique

**Fig. 2:**
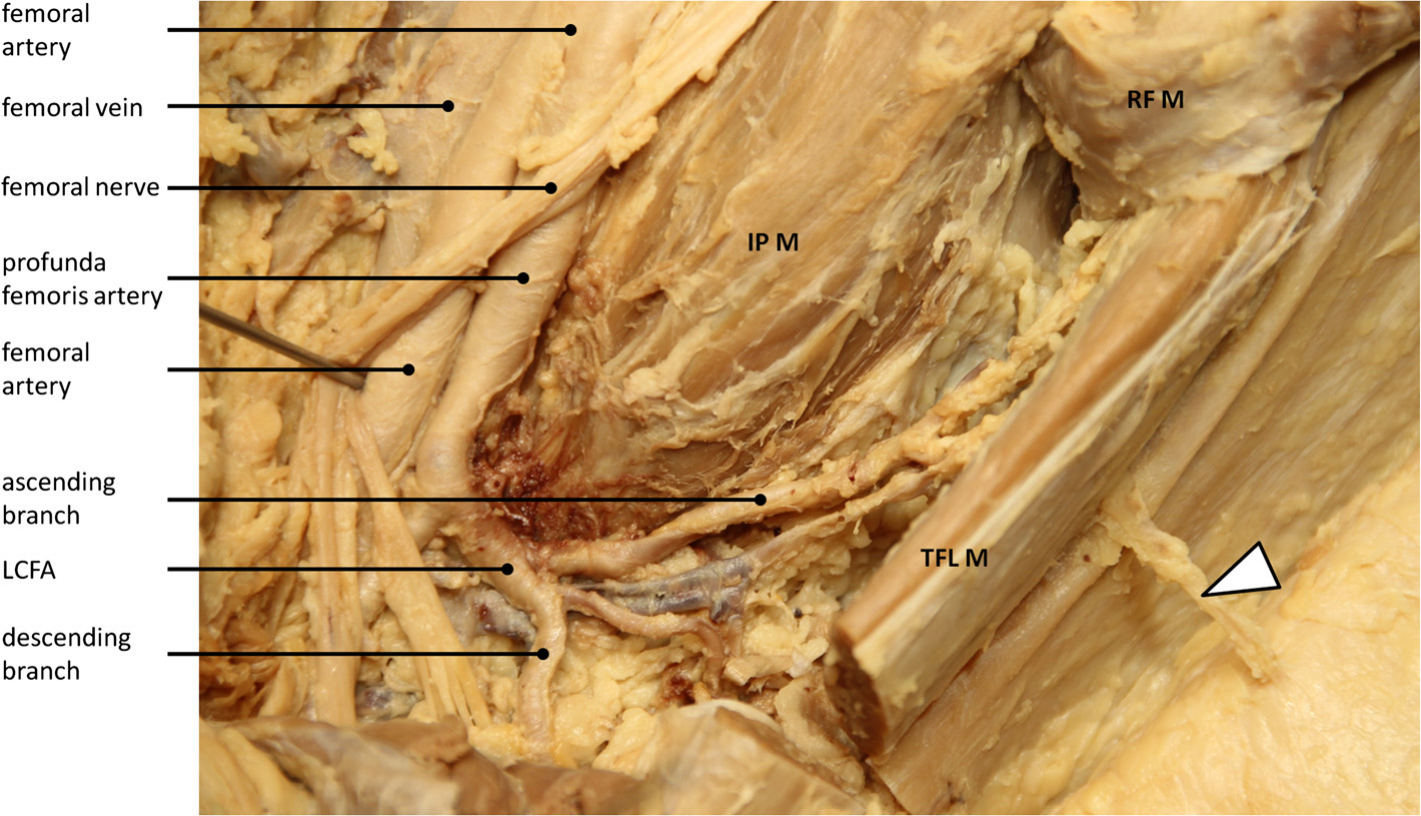
Anatomical dissection of the antero-lateral thigh demonstrating the location of the musculocutaneous tensor fasciae latae perforator (white arrow) at the lateral border of the tensor fasciae latae muscle (TFL M). This perforator emerges from the ascending branch of the lateral circumflex femoral artery (LCFA). The profunda femoris artery, the LCFA and the descending branch of the LCFA are shown at the distal border of the iliopsoas muscle (IP M) after retraction of the sartorius muscle and the rectus femoris muscle (RF M). Note that the femoral nerve and its branches are retracted medially

Septocutaneous and musculocutaneous perforators emerge from the ascending branch of the lateral circumflex femoral artery and can be found at the lateral border of the tensor fasciae latae muscle where they penetrate the subcutaneous tissue [16] (Fig. 2). Such perforators are reported to be constant and are used in plastic surgery to create pedicles for tensor fasciae latae perforator flaps [17, 18]. Anatomical studies have shown that 100 % of the perforators emerge at the lateral border of the tensor between 6 and 15 cm distal from the anterior superior iliac spine [17]. Due to their consistency in appearance and location, these perforators are ideal anatomical landmarks for the identification of the superficial thigh fascia and the tensor fasciae latae muscle when using the minimally-invasive direct anterior approach for total hip replacement.

**Fig. 3:**
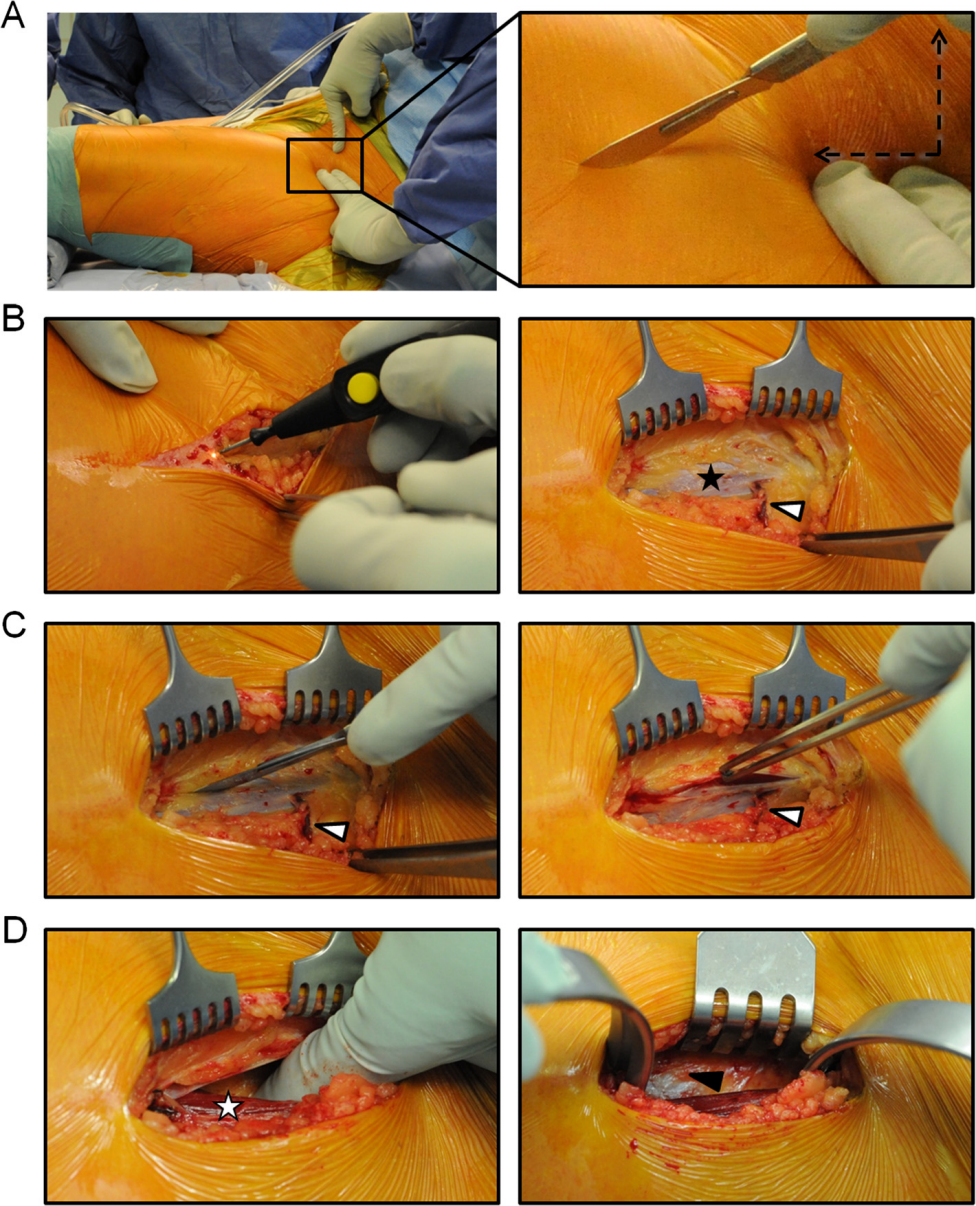
The key surgical steps involved in the minimally-invasive direct anterior approach to the hip are: the correct placement of the skin incision (**a**), the identification of the tensor fasciae latae muscle using the perforator as anatomical landmark (**b**), the incision of the superficial thigh fascia as laterally as possible over the muscle (**c**) and the blunt dissection to enter the Hueter interval (**d**)

The skin incision is placed on an imaginary line between the anterior superior iliac spine and the fibular head. The incision starts two centimetres lateral and distal from the anterior superior iliac spine (Fig. 3a). The superficial subcutaneous fat layer is dissected via monopolar electrosurgical coagulation. The deep subcutaneous fat covering the thigh fasciae (black asterisk) is dissected bluntly using a sterile gauze pad to prevent iatrogenic injury of the lateral femoral cutaneous nerve branches and the tensor fasciae latae perforator (white arrow) is visualized (Fig. 3b). The tensor fasciae latae muscle covered by the superficial thigh fascia can be clearly identified by the perforator (white arrow) that enters the subcutaneous connective tissue (Fig. 3c). The fascia is incised longitudinally at the lateral third of the tensor muscle directly medial to the perforator and separated from the muscle (white asterisk) to enter the Hueter interval (black arrow) (Fig. 3d).

## Discussion

**Fig. 4:**
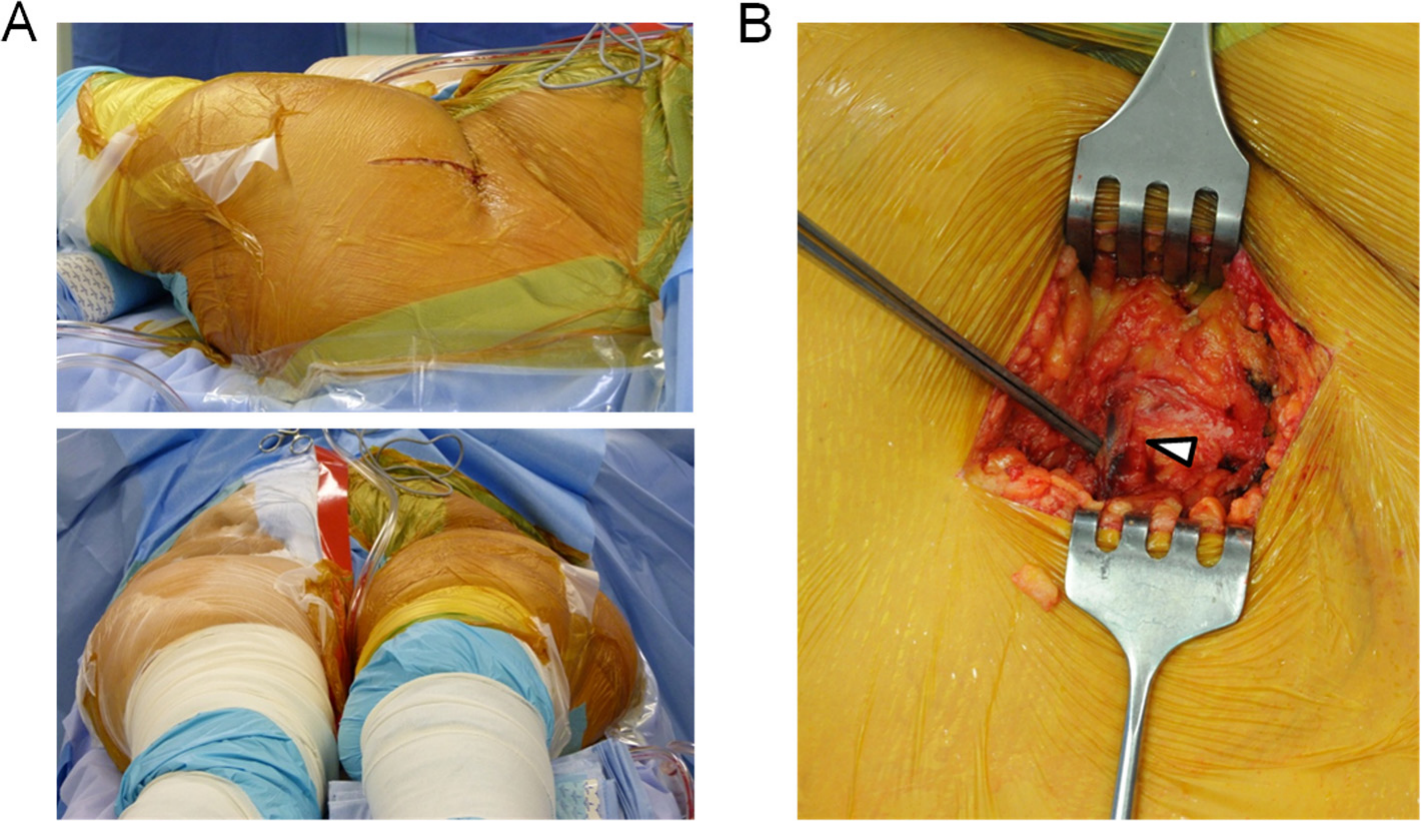
Standardized placement of the skin incision in a 63 year old female patient (BMI > 40 kg/m^2^) undergoing hip replacement via the minimally-invasive direct anterior approach (**a**). Despite the presence of an enormously thick subcutaneous fat layer, the lateral border of the tensor fasciae latae muscle can be easily identified using the tensor fascia latae perforator (*white arrow*) (**b**)

The minimally-invasive direct anterior approach was introduced in our department as the standard approach to the hip joint in 2007 [11]. We and others have demonstrated that this approach results in mid-term and long-term clinical outcomes that are comparable to outcomes following standard procedures such as the lateral transgluteal approach. A shorter hospitalization time, reduced postoperative pain levels, a reduced fatty infiltration rate of the gluteus medius muscle and a shorter skin incision have been identified as specific advantages of this surgical approach [9–11, 19–21]. However, one disadvantage of this approach is a relatively high incidence in lesions of the lateral femoral cutaneous nerve. Damaging this purely afferent sensory nerve can result in a simple hypaesthetic skin area at the lateral thigh, which is usually clinically insignificant, but can also lead to allodynia and eventually a significantly reduced quality of life [4]. To minimize the risk for iatrogenic injury of this nerve, the skin incision and consecutively the incision of the superficial fascia of the tensor fasciae latae muscle should be made as laterally as possible over the belly of the muscle [13–15]. A medial incision is almost inevitably associated with an injury of the main trunk of the nerve. Moreover, in such cases, a later revision and distal extension of the approach poses a very high risk of damaging branches of the femoral nerve [22, 23]. On the other hand, a too far laterally located incision increases the risk of entering the Watson-Jones interval rather than the Hueter interval. As described in our article, a standardized technique for the placement of the skin incision in combination with the use of a constant anatomical landmark such as the tensor fasciae latae perforator to identify the access to the Hueter interval can effectively prevent the above-mentioned complications. Using this technique, even highly adipose patients can be safely treated with total hip replacement via the minimally-invasive direct anterior approach (Fig. 4).

## Conclusion

In the presented technical note, we advocate the use of a constant anatomical structure to clearly identify the Hueter interval and to minimize the risk for iatrogenic nerve injury. As demonstrated, a simple transfer of knowledge between surgical disciplines can significantly advance surgical techniques and eventually improve patient outcomes. The tensor fasciae latae muscle perforator that is routinely used in plastic surgery to lift tensor fasciae latae perforator flaps can serve as a reliable anatomical landmark when using the minimally-invasive direct anterior approach for total hip arthroplasty.
